# FOSTERING IMPROVED OUTCOMES AMONG CARE PARTNERS OF PERSONS LIVING WITH ALZHEIMER’S DISEASE AND RELATED DEMENTIAS

**DOI:** 10.1093/geroni/igae098.1622

**Published:** 2024-12-31

**Authors:** Sara Bybee, Elyse Couch, Darina Petrovsky

**Affiliations:** Chair; Co-Chair; Discussant

## Abstract

The decline in cognitive and functional abilities due to Alzheimer’s Disease and Related Dementias (ADRD) increases the reliance of persons living with dementia (PLWD) on care partners. These care partners, who can be spouses, children, friends, or others, often experience high levels of stress and burden, leading to poor physical and psychological health outcomes. This symposium will feature five speakers who studied ADRD care partner outcomes using various methods across diverse populations. Dr. Darina Petrovsky will discuss findings from the 2017 National Study of Caregiving and National Health and Aging Trends Study, focusing on the relationship between care partner sleep quality, burden, and perceived caregiving benefits. Dr. Shandra Burton will explore cultural factors and resilience among Black/African American care partners using narrative inquiry. Dr. Samantha Shune will present a multiple regression analysis examining the link between PLWD’s dysphagia (swallowing difficulty) and care partner burden. Dr. Elyse Couch will describe a mixed-methods study investigating the impact of care partners receiving PLWD’s positron emission tomography (PET) scan results on their burden. Lastly, Dr. Bybee will review results from an interpretive descriptive analysis of dementia dyads’ open-ended survey responses regarding advance care planning. Dr. Petrovsky will then synthesize the symposium findings and moderate audience questions. Alzheimer’s Disease and Related Dementias Interest Group Sponsored Symposium

